# Molecular identification of novel duck associated Chapparvovirus in ducks, first report from China

**DOI:** 10.1016/j.psj.2025.104984

**Published:** 2025-03-05

**Authors:** Yuanzhuo Man, Weichi Li, Xin Xu, Xiaonan Lu, Dandan Li, Lunguang Yao, Jun Ji, Yingzuo Bi, Qingmei Xie

**Affiliations:** a Henan Provincial Engineering Laboratory of Insects Bio-reactor, Henan Provincial Engineering, and Technology Center of Health Products for Livestock and Poultry, Henan Provincial Engineering and Technology Center of Animal Disease Diagnosis and Integrated Control, Nanyang Normal University, Nanyang 473061, PR China; b College of Animal Science, South China Agricultural University, Guangzhou 510642, PR China

**Keywords:** Duck Associated Chapparvovirus, Chaphamparvovirus, Recombination, Evolution analysis, Mutation sites

## Abstract

Duck Associated Chapparvovirus (DaChPV) is a newly discovered virus within the Chaphamparvovirus genus, and first identified from Canadian wild ducks in 2021. In this study, DaChPV DNA was detected in 14 out of 137 tissues samples collected from diarrhea ducks across various provinces in China. Subsequently, eight complete genome sequences were amplified using overlapping primers and sequenced. Comparative analysis revealed that the amino acid (aa) sequences of NS1 and VP1 from these eight DaChPV strains shared identity with reference strains, ranging from 81.43 % to 98.81 % for NS1 and 76.95 % to 98.39 % for VP1, respectively. Phylogenetic analysis of the genome sequences showed that the newly identified DaChPVs and reference DaChPV strains formed an independent cluster, indicating a close genetic relationship. The strains of AH2301, HN2201 and HN2301 identified in this study were classified as belonging to Duck Associated Chaphamapavovirus 1. In contrast, AH2401, HN2401, and SD2301 were grouped with Duck Associated Chaphamapavovirus 2. The remaining two strains, HeB2201 and HeB2401, may represent variant strains that cluster independently, which is further supported by the evolutionary tree results of VP1 and NS1. Additionally, inter-type recombinations were predicted for these DaChPV strains. The contained multiple specific mutation sites, including 63, 76, 78, 303, and 305 that located on the predicted antigenic epitopes. This research firstly determined the evolutionary trends of DaChPV in China, offering valuable insights for understanding of spread, evolution, and molecular epidemiology of DaChPV on a global scale.

## Introduction

Parvoviruses are a type of small, non-enveloped icosahedral viruses composed of single-stranded DNA genomes with about 4 – 6 Kb in length (Cotmore and Tattersall, 2014; Tijssen, et al., 2016). Recent advancements in molecular techniques, such as high-throughput sequencing and high-sensitivity PCR, have enabled identification of numerous novel parvoviruses (de Souza, et al., 2018; Palinski, et al., 2016). Currently, Parvoviruses have been divided into three subfamilies: *Parvovirinae* (11 genera) infect vertebrates; *Densovirinae* (11 genera) infect invertebrates; *Hamparvovirinae* contains members identified from both vertebrate (2 genera) and invertebrate hosts (3 genera) (Pénzes, et al., 2020). Although the *Chaphamaparvovirus* genus was only recently established, several microorganisms from this genus have been identified in various hosts, such as mice (Yang, et al., 2016), bats (Yinda, et al., 2018), pig (Palinski, et al., 2016), and black-necked cranes (Li, et al., 2020; Zhao, et al., 2023). In 2022, Chicken Chapparvovirus (CkChpV) DNA was detected in 7 out of 121 healthy chickens and 116 out of 357 chickens exhibiting diarrhea. The obtained 9 complete genome sequences of the CkChpV strains also confirmed the presence of CkChpV infection in Chinese chickens (Cui, et al., 2023).

Parvoviruses have significantly impacted poultry-related industries globally, with various types of Parvoviruses identified in different poultry hosts. Among them, goose parvovirus (GPV) and muscovy duck parvovirus (MDPV), are both classified under *Anseriform dependoparvovirus 1*, cause high mortality rates in waterfowls, ranging from 10 % to 80 % (Chen, et al., 2015). GPV, the pathogen responsible for Derzsy's disease, manifests in symptoms such as drowsiness, growth retardation, anorexia, motor dysfunction and watery diarrhea (Soliman, et al., 2020). Even though MDPV share relative high genome similarity with GPV, it only results in fatal diseases in young Muscovy ducks, distinguishing it from GPV (Wan, et al., 2019). Short beam and dwarfism syndrome (SBDS) affecting mule ducks was originally believed to be caused by infection of the West European lineage of GPV. However, since 2014, a novel GPV-related parvovirus (N-GPV) has emerged in China, and can also induce the infected commercial Cherry Valley ducks to develop SBDS (Chen, et al., 2016; Yu, et al., 2016). In addition to common symptoms like SBDS, N-GPV can also cause the infected waterfowls suffering delayed growth, watery diarrhea, mild liver detachment and slight thymic bleeding (Palya, et al., 2009; Zhu, et al., 2022).

Recently, another novel parvovirus namely Duck Associated Chapparvovirus (DaChPV) was initially identified in wild ducks from Canada in 2021, has been classified under the genus *Chaphamparvovirus* within the subfamily *Hamamaparvovirus*. DaChPVs possess two open reading frames encoding non-structural protein (NS1, 674 amino acid (aa)) and capsid protein (VP1, 540 aa), respectively. Through the evolution tree generated using the NS1 aa sequences of the Chaphamapavovirus genus, the five novel DaChPVs clustered closed but categorized into two sub-branches by ICTV in 2023: Duck Associated Chaphamapavovirus 1 (DaChPV1, classified as *Chaphamapavovirus anseriform5*) and Duck Associated Chaphamapavovirus 2 (DaChPV2, classified as *Chaphamapavovirus anseriform6*) (Canuti, et al., 2021). Apart from this, the infection and pathogenic mechanisms of DaChPV are still unclear.

To enhance our understanding of the infection status of DaChPV in China, we gathered and screened tissue samples from both ducks with or without diarrhea that sourced from several representative poultry farming province of central and eastern China.

## Materials and methods

### Sample collection and ethics approval

In this study, intestinal tissue samples were collected from 318 naturally dead ducks (181 ducks without apparent clinical symptoms and 137 ducks with diarrhea), that obtained from poultry farms in Henan, Hubei, Hunan, Hebei, and Anhui Provinces from 2020 to 2024. The ducks without apparent clinical symptoms might die due to stress or other husbandry management errors. The research adhered to the ethical standards for animal experimentation set by Nanyang Normal University.

### Nucleic acid extraction and pathogen screening

Collected tissue samples were processed by freezing and milling into powder in liquid nitrogen. Easy Pure Viral DNA/RNA Kit (Trans Gen Biotechnology, Beijing, China) was used to extract viral DNA/RNA from the tissue powder. Primers for semi-nested PCR (snt-PCR) were designed to detect DaChPV, utilizing two pairs of primers in the first and second rounds, which include DaChPV-FIO/DaChPV-RO and DaChPV-FIO/DaChPV-RI, as shown in Table 1. Snt-PCR was conducted using the Taq Plus Master Mix kit (Vazyme Biotech Co., Ltd, Nanjing, P. R. China). The protocol included an initial pre-denaturation stage at 95°C for 5 minutes, followed by 35 cycles of denaturation at 95°C for 30 seconds, annealing at 50°C (outer amplicons) or 52°C (inner amplicons) for 30 s, and extension at 72°C for 40 s, with a final extension at 72°C for 10 m. Additionally, PCR/RT-PCR techniques were also employed to detect six other pathogens in the samples, including Duck associated ambidenovirus1 (DaaDV1), Newcastle disease virus (NDV), goose astrovirus (GoAstV), duck enteritis virus (DEV), duck circovirus (DCV) and GPV. The sequences of primers employed in all screening experiments were presented in Table 1. The infection status for various viruses were visualized using a Venn diagram (http://jvenn.toulouse.inra.fr/app/example.html) and an UpSet diagram generated by TBtools-II version (Chen, et al., 2023).

**Table 1:** Primers used for virus detection in this study

Primer name	Primer Sequence (5′–3′)	Reference
**DaChPV-OIF**	GGAYTWGGWAAGTGYTGTCC	This study for nested PCR
**DaChPV-OR**	GTCCTTYTTGATTHKGACACC	
**DaChPV-IR**	GTGTNCKWGGTAACATATAYGG	
**DaChPV-F1**	GCCTGGAGGTACTCCAGAAGA	This study for genome amplification
**DaChPV-R1**	TCCAAGTCCTRCGAAWARGCT	
**DaChPV-F2**	GCTCCAGAKGTSGTTGTGA	
**DaChPV-R2**	AGCCCATTGTCTWGGTGTWAKT	
**DaChPV-F3**	GATTGGGCGGGAARATGGA	
**DaChPV-R3**	TTGCTCGTTTRTTTGTWKSTGTG	
**DaChPV-F4**	CTGGKCAGGMACAGAATGG	
**DaChPV-R4**	GGCCGCGTTACGCAATAAAG	
**DaaDV1-OF**	CTCTCCCATAGGAACATTTCC	(Canuti, et al., 2021)
**DaaDV1-OIR**	GGAGTACAACCAGTTCCAGC	
**DaaDV1-IF**	GCGTAAGGCCATGCGGTTGG	
**NDV-F**	GGAGGATGTTGGCAGCATT	(Pang, et al., 2002)
**NDV-R**	GTCAACATATACACCTCATC	
**DEV-F**	GAAGGCGGGTATGTAATGTA	(Zhu, et al., 2022)
**DEV-R**	CAAGGCTCTATTCGGTAATG	
**GPV-F**	CAATGGGCTTTTACCAATATGC	
**GPV-R**	ATTTTTCCCTCCTCCCACC	
**DCV-F**	CCCGCCGAAAACAAGTATAA	
**DCV-R**	TCGCTCTTGTACCAATCACG	
**GoAstV-F**	TGGTGGTGYTTYCTCAARA	(Yang, et al., 2018)
**GoAstV-R**	GYCKGTCATCMCCRTARCA	

### Entire genome sequencing

Based on the genome sequences of DaChPV strain (BE7) and various reference strains of DaChPV, four pairs of primers were designed and synthesized to amplify overlapping sequences of DaChPV gene fragments. A reaction mixture of 20 μL was prepared for PCR, which included template DNA (above 100 ng/μL), upstream and downstream primers, Green-Taq Mix (Vazyme Biotech Co., Ltd, Nanjing, P. R. China) and ddH_2_O. The cycling conditions for amplification included pre-denaturation at 94°C for 3 m, totaling 35 cycles of denaturation at 94°C for 30 s, annealing at the optimal temperature of the primers for 30 s, extension at 72°C for 1 m and 20 s; and finally ending with last extension at 72°C for 10 minutes. The purified amplified products were subsequently cloned into the pMD18-T vector (TaKaRa Bio Co., Ltd., Dalian, China) and sequenced by GENERAL BIOL (Chuzhou, China).

### Sequence identification and phylogeny

Whole genome nucleotides (nts), along with the NS1 and VP1 aa sequences of the DaChPVs, Chaphamaparvovirus anseriform1 (MT247758, CTCPaV1/CT08.18-AU-2018), Chaphamaparvovirus anseriform2 (MT247759, CTCPaV2/CT08.18/12952-AU-2018), Chaphamaparvovirus anseriform3 (MT247760, CTCPaV3/CT08.18/12952-AU-2018), as well as other parvoviruses obtained from GenBank database, were aligned via the Clustal W algorithm of DNAStar 7.1 (Madison, WI, USA). The sequence similarities were processed using ChiPlot (https://www.chiplot.online/) to display. Using the Tamura-3 parameter model and maximum likelihood method, MEGA11 (version 11.0.10) software, were employed to constructing phylogenetic trees for the genome nt, NS1 and VP1 aa via 1000 bootstrap repeating (Tamura, et al., 2021). Finally, phylogenetic trees were further annotated and visualized using web-based tools (https://www.chiplot.online/).

### Recombinant analysis

RDP v-4.36 was employed for recombination prediction across the eight DaChPVs’ genome refer to the reference strains. To ensure the accuracy, seven distinct recombination analysis algorithms were utilized, composed of RDP, GENECONV, Bootscan, Maxchi, Chimaera, SiSscan, PhylPro, LARD and 3Seq (Martin, et al., 2015). Afterwards, the prediction events were further checked and illustrated utilizing SimPlot 3.5.1.

### Analysis of mutations and antigenic epitopes

Mutation sites harbored in NS1 and VP1 proteins across the eight DaChPV strains were compared with reference strains of BE7 (MW306775, DaChPV1), B6 (MW306774, DaChPV1), B55 (MW306776, DaChPV2), and BE8a (MW306778 DaChPV2). Additionally, DNAMAN Version 5.2.2 (Lynnon Biosoft, Canada) was employed to predict and analyze the predominant antigenic epitopes present in the VP1 protein of DaChPVs.

### Statistical analysis

The fisher's exact test was employed using GraphPad Prism 10.1 (La Jolla, CA, USA) to compare the DaChPV frequency between ducks without apparent clinical symptoms and diarrheic ducks. A *P* <0.05 was considered statistically significant.

## Results

### Pathogen investigation

**Fig. 1:**
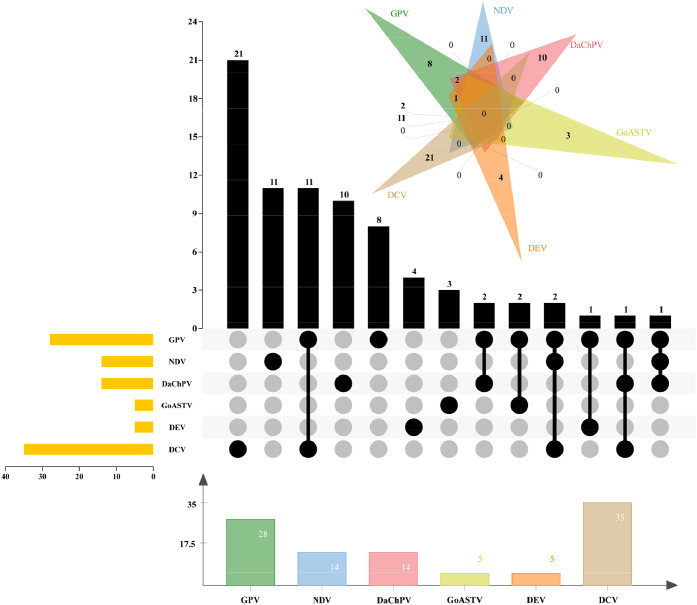
The distribution of DaChPV in China, along with the positive rates of GPV, NDV, GoASTV, DEV and DCV, is illustrated through a combination of a Venn diagram and an UpSet plot. The UpSet plot displays the distribution of various viruses across the samples.

Based on snt-PCR detection results, 14 out of 137 (10.2 %) diarrheic ducks and 1 out of 181 (0.6 %) ducks without diarrhea were tested positive for DaChPV. Fisher's Exact Test (*P* <0.05) indicated associations between DaChPV and diarrhea symptom. And in the screened diarrhea duck samples, the positive rates of DaaDV1, GPV, NDV, GoAstV, DEV, and DCV were 0 % (0/137), 20.4 % (28/137), 10.2 % (14/137), 3.6 % (5/137), 3.6 % (5/137), and 25.5 % (35/137), respectively (Fig. 1). Among the DaChPV positive samples, 10 samples were found to be infected with DaChPV alone, while an additional 4 ones were co-infected with GPV, DCV or NDV. The screening results of the pathogenic microorganisms are illustrated in Fig. 1.

### Sequence and phylogenetic analysis

**Fig. 2:**
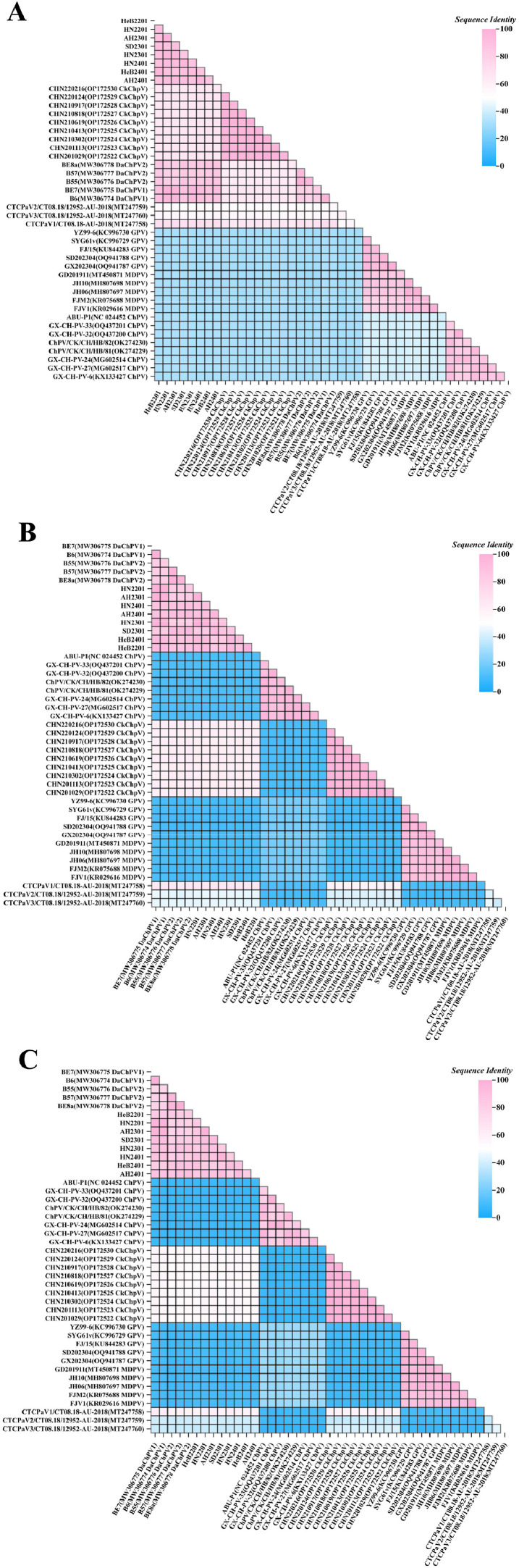
Sequence identity between DaChPV and DaChPV reference strains. (A) Genome wide sequence identity. (B) Sequence Identity of NS1. (C) Sequence Identity of VP1.

The obtained genomes of the eight DaChPV strains (consist of 1 from ducks without diarrhea and 7 from diarrhoeal ducks) was approximately 4230 nt in length, and under the accession numbers PQ602465 ∼ PQ602472 in the GenBank database. These 8 genomes mainly encoded two proteins of NS1 and VP1, which were similar to the reference DaChPVs. The genomic nt identity between the eight DaChPV strains and the initially reported reference DaChPV strains ranged from 79.0 % (HN2201 and B57 (DaChPV2, accession NO.: MW306777)) to 97.9 % (HN2201 and BE7 (DaChPV1, accession NO.: MW306775)). Compared with CkChpV genomes, the sequence identity ranged from 65.3 % (HeB2201 and CHN220216 (CkChpV, accession NO.: OP172530) to 66.0 % (SD2301 and CHN210917 (CkChpV, accession NO.: OP172528). Furthermore, in comparison to MDPV, GPV and other Chapparvovirus, the sequence identity ranged from 30.7 % (HN2401 and FJM2 (MDPV, accession NO.: KR075688)) to 70.5 % (AH2301 and CTCPaV1/CT08.18-AU-2018 (Chaphamapavovirus anseriform1, accession NO.: MT247758)). The NS1 protein sequences of the eight DaChPV strains exhibited similarities ranging from 81.4 % (between AH2301 and B57, DaChPV2, access NO.: MW306777) to 98.8 % (between HN2201 and BE7, DaChPV1, access NO.: MW306775) when compared to the DaChPV reference strains. Similarly, the VP1 aa similarity with the DaChPV reference sequences ranged from 77.3 % (between HN2201 and B55, DaChPV2, access NO.: MW306776) to 98.4 % (between AH2301 and BE7, DaChPV1, access NO.: MW306775). The CTCPaV1/CT08.18-AU-2018 strain (Chaphamapavovirus alternative 1, access NO.: MT247758) demonstrated relatively higher similarity to the DaChPV strain, with 65.1 % NS1 aa similarity (compared with AH2401) and 60.5 % VP1 aa similarity (compared with AH2301), in comparison to other non-DaChPV reference strains. In contrast, strain FJ/15 (GPV, access NO.: KU844283) and GX-CH-PV-33 (Chicken parvovirus, access NO.: OQ437201) exhibited lower similarity, with 9.9 % NS1 aa similarity (between SD2301 and FJ/15) and 8.0 % VP1 aa similarity (between HeB2201 and GX-CH-PV-33), respectively (Fig. 2). DaChPV exhibits low similarity with other virus reference sequences outside the Chaphamparvovirus genus, likely attributable to distant phylogenetic relationships.

**Fig. 3:**
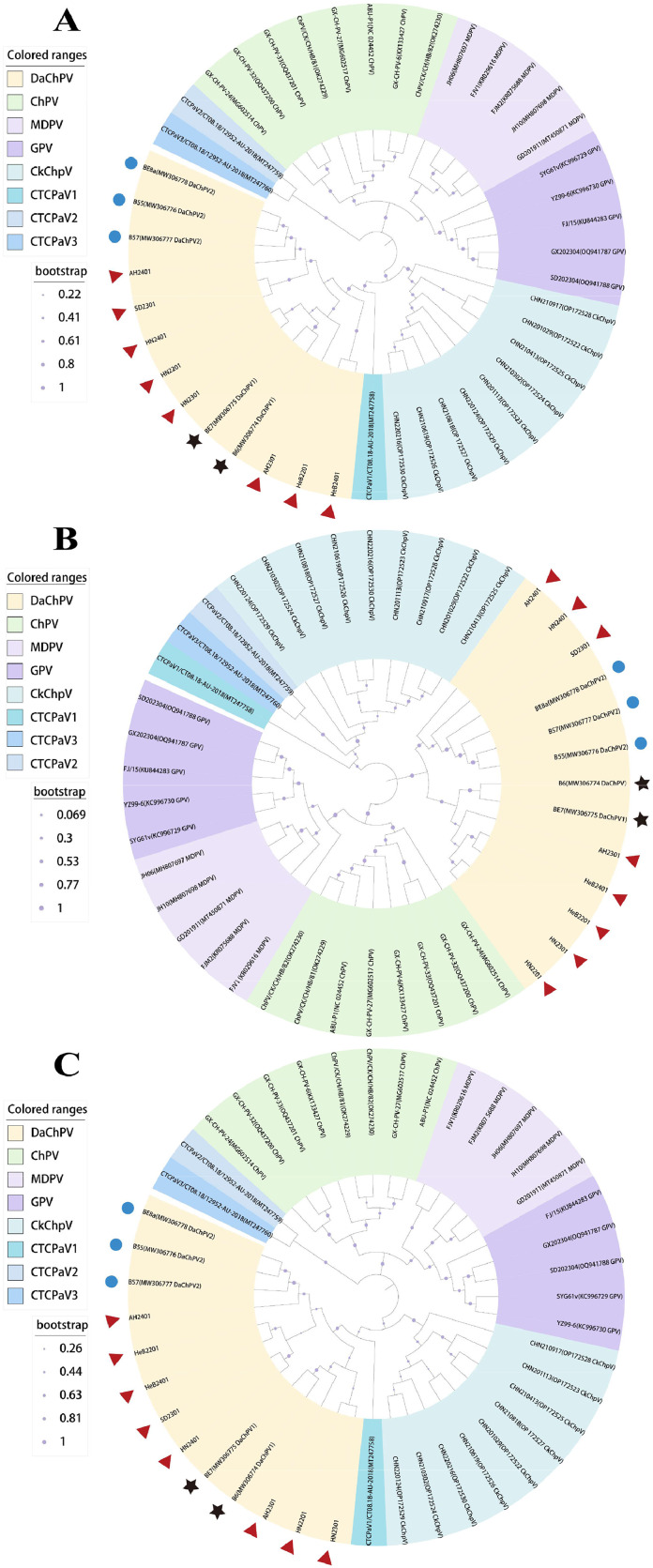
Phylogenetic tree of DaChPV whole genome, NS1 and VP1 genes. The eight DaChPV detectors in this report are indicated by solid orange stars. The same color is used to identify species. (A) Whole genome phylogenetic tree. (B) Phylogenetic tree of NS1. (C) phylogenetic tree of VP1; The strains marked with red triangle were detected in this experiment. The strain marked with black pentagram is the DaChPV1 reference strain; The strains marked with blue circle are DaChPV2 reference strains.

Phylogenetic analysis of the genome sequences revealed that the 8 obtained and the 5 reference DaChPV strains, and other strains within the Chaphamparvovirus genus (including CkChpV and Chaphamapavovirus anseriform1) clustered into one larger branch. Furthermore, similar to the phylogenetic tree of the entier genome, AH2301, HN2201 and HN2301 formed a sub-branch with DaChPV1, while AH2401, HN2401 and SD2301 formed a sub-branch with DaChPV2 in the aa evolutionary tree of VP1 and NS1. However, HeB2201 and HeB2401 strains differed from the others, as they formed a small branch separately in the genome evolutionary tree. Differently, they formed sub-branches closed to DaChPV1 in the NS1 evolution tree and DaChPV2 in the VP1 evolutionary tree, respectively (Fig. 3).

### Recombination analysis

**Fig. 4:**
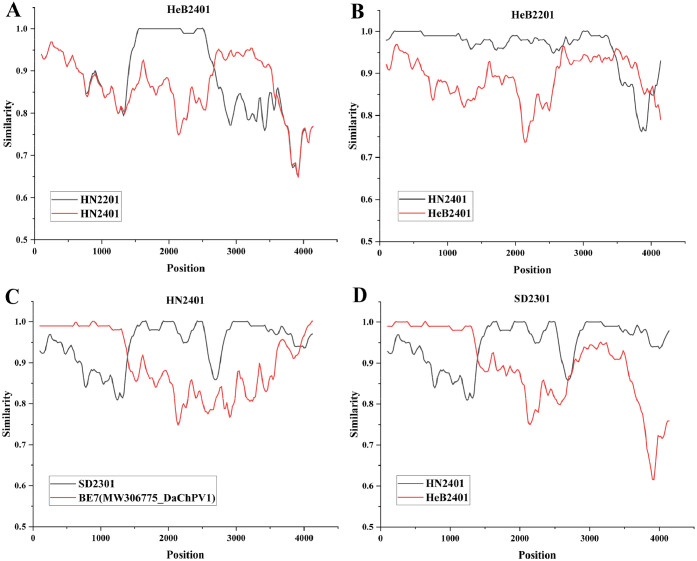
Representative recombinant strains and predicted recombination events. The recombination events representing the strains were predicted using SimPlot software, with the intersection of the two lines indicating the recombination breakpoints.

As predicted by RDP4 and recalculated using SimPlot, a comprehensive list of the main recombination events associated with the obtained DaChPV strains were presented in Table 2. The recombination event suggested that HeB2401 may have originated from the recombination involving the minor parent (HN2201) and the major parent (HN2401). HeB2201 is a recombinant of strains HN2401 and HeB2401. Events 1 and 2 indicate the presence of inter-type recombination in DaChPV, while Event 3 implies the potential for recombination between domestic and foreign strains (Fig. 4).

**Table 2:** Recombination analysis of the DaChPV strains included in this study

Recombination strains	Breakpoint positions		Minor parent		Major parent		*p*-value
	Start	End		Similarity		Similarity	
HeB2401	1429	2629	HN2201	89.16 %	HN2401	85.12 %	8.06E-30
HeB2201	3557	4122	HN2401	89.63 %	HeB2401	96.01 %	3.26E-03
HN2401	1422	3528	SD2301	95.18 %	BE7	91.5 %	9.35E-09
SD2301	3542	4229	HN2401	95.18 %	HeB2401	90.35 %	8.26E-23

### Analysis of aa mutations and antigenic epitopes

The VP1 protein of the 13 DaChPV strains underwent B-cell epitope prediction, leading to the identification of 10 potential dominant antigenic epitopes. Notably, the epitope located at residues 363-381 exhibited the highest score of 1.169 (Table 3).

**Table 3:** VP1 epitope location and sequences

Epitopes	Residues (amino acid)	Epitope sequences	Score
A	363–381	CYTYPPCQWFIKGIPLCDA	1.169
B	431–439	IFQPACIRY	1.147
C	299–308	PSGVVAYEDY	1.134
D	129–139	NKQLHLALKEG	1.123
E	46–60	HIIPNFLWRHVLTPR	1.121
F	62–105	WAAFVTECEAYQIKSIHGTVYNPIP ITTNLSLQRVNLFSAFNNC	1.111
G	281–289	AFACTYGLA	1.109
H	389–399	TTQVSFRISIT	1.096
I	16–30	DNAFFQYPSIDVETI	1.093
J	261–272	GPYCGVGRPYEL	1.092

Compared to reference DaChPV1/2 strains, the mutation sites in NS1 and VP1 protein of the 8 DaChPV strains were summarized. Mutations sited at 356, 431, 484, and 626 in NS1 were only harbored in strains of HN2401, SD2301 and HeB2401. Meanwhile, high-frequency mutations in VP1 of the DaChPV strains were sited at 63, 76, 78, 303 and 305. The HeB2201 sequence demonstrates the continuous mutations within the 542aa - 559aa region at the terminal end of the VP1. The remaining mutations in NS1 and VP1 are detailed in the Table 4a and 4b.

**Table 4a:** This study analyzed the major aa mutation sites in the DaChPV NS1 protein of identified and reference DaChPV strains

NS1 Position	BE7	B6	B55	B57	BE8a	HN2201	AH2301	HN2401	AH2401	HN2301	SD2301	HeB2401	HeB2201
135	S	S	S	S	S	S	S	S	S	S	N	N	N
273	S	S	K	K	K	N	S	N	N	N	K	K	K
284	Y	Y	S	S	S	H	Y	H	Y	H	S	S	Y
298	D	D	H	H	H	D	N	D	D	D	Q	Q	Q
320	F	F	F	F	F	F	F	F	F	F	F	Y	Y
356	E	E	E	E	E	E	E	D	E	E	D	E	E
430	E	E	L	L	L	E	E	T	L	E	T	E	E
431	Y	Y	Y	Y	Y	Y	Y	F	Y	Y	F	Y	Y
439	V	V	R	R	R	V	I	H	R	V	H	V	I
443	I	I	S	S	S	I	I	T	S	I	T	I	I
464	S	S	C	C	C	S	S	C	C	S	C	S	G
482	A	A	A	A	A	A	A	V	V	A	V	A	A
484	G	G	G	G	G	G	G	R	G	G	R	G	G
485	N	N	H	H	H	N	N	D	H	N	D	N	N
515	R	R	R	R	R	G	G	R	R	G	R	G	G
521	A	A	T	S	T	A	T	T	T	A	T	A	T
548	V	V	V	V	V	V	I	V	V	V	V	V	I
560	Q	Q	S	S	S	Q	Q	Y	S	Q	Y	Q	Q
570	T	T	I	T	I	T	I	S	I	T	S	T	I
581	G	G	S	S	S	G	G	T	S	G	T	G	G
582	G	G	G	G	G	G	R	G	R	G	G	G	R
588	D	D	D	D	D	N	D	D	D	N	V	N	D
593	S	S	S	S	S	S	S	S	S	S	T	S	S
601	T	T	T	P	T	T	T	N	N	T	H	T	T
619	V	V	V	V	V	V	I	V	V	V	I	V	V
622	S	S	E	E	E	S	S	K	K	S	E	S	S
626	L	L	L	L	L	L	L	V	L	L	V	L	L
631	D	D	D	D	D	D	D	A	D	D	A	D	D
634	K	K	K	K	K	K	K	T	K	K	T	K	K
654	N	N	R	R	R	N	N	R	Q	N	R	N	N

**Table 4b:** Analysis of major aa mutation sites in the VP1 protein of the identified and reference DaChPV strain

VP1 Position	BE7	B6	B55	B57	BE8a	HeB2201	HN2201	AH2301	SD2301	HN2301	HN2401	HeB2401	AH2401
21	Q	Q	K	K	K	Q	Q	Q	H	Q	H	Q	Q
32	I	I	L	L	L	I	V	I	L	V	L	V	L
36	P	P	K	K	K	P	A	P	P	A	P	A	P
38	A	A	A	A	A	A	A	A	S	A	S	A	A
63	A	A	A	A	A	V	A	A	A	A	V	V	A
76	S	S	S	S	S	A	S	S	S	S	A	A	S
78	H	H	H	H	H	K	H	H	H	H	K	K	H
124	Y	Y	F	F	F	H	Y	Y	H	Y	H	H	F
143	T	T	T	T	T	S	T	T	T	T	T	S	T
145	N	N	A	A	A	A	N	N	Y	L	Y	A	A
146	-	-	M	M	M	M	-	-	D	Y	D	M	M
147	M	M	T	T	T	T	M	M	N	N	N	T	T
148	Q	Q	Q	Q	Q	T	Q	Q	K	N	K	T	Q
149	S	S	E	E	E	S	S	S	R	H	R	S	E
150	S	S	S	S	S	G	S	S	Q	Q	Q	G	S
151	S	S	S	S	S	S	A	S	S	S	S	S	S
152	G	G	G	G	G	G	G	G	V	G	V	G	G
153	S	S	S	S	S	G	S	T	S	S	S	G	S
154	-	-	-	-	-	E	-	-	-	-	-	E	-
188	T	T	T	T	T	T	V	V	V	V	V	T	V
203	A	A	S	S	S	A	S	S	T	S	T	A	S
242	S	S	E	E	E	D	S	S	E	S	E	D	E
245	E	E	K	K	K	Q	E	E	Q	E	Q	Q	K
249	Y	Y	Y	Y	Y	Y	Y	Y	F	Y	F	F	Y
253	Q	Q	Q	Q	Q	V	Q	Q	V	Q	V	V	Q
274	E	E	E	E	E	E	E	Q	E	E	E	E	E
287	C	C	T	T	T	T	S	T	T	S	T	T	T
303	S	S	S	S	S	A	S	S	S	S	S	A	S
305	V	V	V	V	V	N	V	V	N	V	N	N	V
340	E	E	Q	Q	Q	A	E	E	A	E	A	A	E
342	T	T	T	T	T	L	T	T	L	T	L	L	T
343	G	G	G	G	G	T	G	G	S	G	S	T	G
344	N	N	-	-	-	N	N	N	N	N	N	-	K
345	T	T	K	K	K	N	T	T	N	T	N	N	N
346	A	A	N	N	N	T	A	A	T	A	T	N	T
347	D	D	T	T	T	-	D	D	-	D	-	T	-
348	A	A	A	A	A	A	A	A	E	A	E	E	A
366	C	C	C	C	C	A	C	C	C	C	A	C	C
400	S	S	S	S	S	C	S	S	S	S	S	S	S
452	Q	Q	Q	Q	Q	Q	Q	Q	R	Q	Q	Q	Q
463	A	A	S	S	S	T	A	T	T	S	V	S	V
487	W	W	W	W	W	W	R	W	W	W	W	W	W
497	V	V	C	C	C	T	V	T	T	C	V	C	V
511	A	A	A	A	A	A	A	A	A	P	A	P	A
546	R	R	Y	I	I	N	R	R	R	I	R	K	R
547	P	P	R	P	P	D	P	P	P	P	P	P	P
548	E	E	N	K	K	D	E	E	E	K	E	K	E
552	R	R	E	R	R	H	R	R	R	R	R	R	R
553	K	K	K	K	K	Q	K	K	K	K	K	K	K
554	S	S	Q	A	A	E	S	S	S	A	S	A	S
555	G	G	A	S	S	N	G	G	G	S	G	S	G
556	S	S	Q	S	S	-	S	S	S	S	S	S	S
557	I	I	Y	I	I	-	I	I	I	I	I	I	I
558	S	S	Q	S	S	L	S	S	S	S	S	S	S
559	K	K	S	K	K	E	K	K	K	K	K	K	K
560	L	L	C	L	L	A	L	L	L	L	L	L	L
561	L	L	/	L	L	Y	L	L	L	L	L	L	L
563	L	L	/	L	L	S	L	L	L	L	L	L	L
564	H	H	/	H	H	C	H	H	H	H	H	H	H

## Discussion

Recently, the intensive poultry farming system promotes the development of the poultry industry, whilst also has been a significant factor contributing to the emergence of new pathogens over the last two decades (Delabouglise, et al., 2020; Gržinić, et al., 2023). In this study, DaChPV was firstly identified in Chinese domesticated ducks, with a positive rate of 4.7 % (15/318), following its discovery in wild ducks in Canada (Canuti, et al., 2021). The identification of DaChPV in domestic duck populations in China, that implied the potential for its widespread occurrence in other duck populations globally and the potential threat of the DaChPV to the global duck industry. The positivity rate of DaChPV in ducks with diarrhea was 10.2 % (14/137), statistical analysis had shown that DaChPV infection was correlated with clinical symptoms (*P* < 0.05). In the diarrhea samples positive for DaChPV, we identified four ones co-infected with DCV, GPV and NDV. Infections of GPV and NDV are known to produce diarrhea symptoms. Therefore, further experimental verification was necessary to ascertain whether DaChPV alone can induce diarrhea. We have attempted to isolate DaChPV by duck embryos and detect its presence using PCR. However, no DaChPV was detected in yolk sacs, allantoic fluid and internal organs. According to the investigation results, no samples were positive for DaaDV1, that belonged to the *Densovirinae* subfamily and Protoambidensovirus genus and had also been identified alongside DaChPV in North American ducks (Canuti, et al., 2021).

As indicated by the phylogenetic analysis, both DaChPV1 and DaChPV2 are currently prevalent in China. With the exception of the six DaChPVs whose genotypes can be clearly defined, the strains HeB2201 and HeB2401 have not yet undergone subtyping and are temporarily designated as DaChPV due to the complexities inherent in the existing data. Notably, HeB2201 and HeB2401 occupied the same branch as DaChPV1 in the NS1 evolutionary trees and DaChPV2 in the VP1 evolutionary. In terms of VP1 aa sequences, strain HeB2201 exhibited an 84.8 % similarity to DaChPV1, which was at odds with the findings from the phylogenetic analysis. This discrepancy may result from recombination events that altered the VP1 sequence of HeB2201. The predictions indicated that HeB2201 might be a recombinant strain derived from HN2401 and HeB2401. This peculiar phenomenon suggested the complex evolutionary direction and rate of prevalent strains in different regions. Despite the trade of poultry between different provinces, the genetic evolution of strains across different regions appeared to be relatively independent. Meanwhile, the recombination analysis conducted in this study was based on available sequence fragments which were for reference only, which might not objectively represent the actual recombination event. Refer to recombination prediction results, HeB2201 exhibited a recombination breakpoint within the coding region of the DaChPV VP1 protein, between nucleotides 3557 and 4122, which showed greater similarity to the DaChPV1 strain of HN2401. Additionally, predictions of recombination event indicated that HeB2401 might undergo inter-type recombination, with breakpoints located at positions 1429 and 2629, which s encompassed portions of the NS1 and VP1 coding sequences, potentially affecting its evolution and host infection capabilities. In summary, strains of HeB2201 and HeB2401 may represent evolution trend of the new subtype of DaChPV. Refer to the recombination event illustrated in Fig. 4, there were other recombination events, indicating that the recombination process associated with the epidemic DaChPV strain was more complex than initially thought.

Additionally, among the 15 DaChV positive samples, we successfully obtained complete sequences for only 8 strains of DaChV, while complete sequences for the remaining samples were not obtained. We speculate that more significant mutations occurred in the missing regions. Finally, we identified the aa mutation sites of eight DaChPV strains’ NS1 and VP1. Multiple mutation sites in VP1 were observed, specifically at positions of 63, 76, 78, 303, and 305, which are located on the predicted antigenic epitopes and may provide valuable data to support future antibody preparation. As the capsid protein and sole antigenic protein of DaChPV, VP1 can stimulate the host's immune system to produce neutralizing antibodies, thereby exerting anti-infective effects (Pénzes, et al., 2020). The mutations identified at multiple sites within VP1 in this study may influence the virus's capacity to infect the host. The aa mutation sites in NS1 are fewer compared to those in VP1. For NS1 is a replication initiator protein located in the nucleus, which would be more conserved because its critical role in the replication and packaging of viral DNA (Cotmore, et al., 2019).

In summary, this study offers the initial account of the identification of DaChPV in domestic waterfowl and elucidates its evolution, recombination, representative mutation sites, and potential antigenic epitopes. These findings serve as a reference for understanding the spread and evolution of DaChPV. Given that DaChPV may have a high prevalence and infectivity, further studies are needed to confirm the pathogenicity of DaChPV and to provide insights into the molecular epidemiology of DaChPV on a global scale.

## Explanation of author contributions

JJ and XX conceived and designed this study; JJ and XNL conducted sampling and data collection; YZM performed clinical research and molecular testing, while WCL conducted research in molecular genetics; XNL and DDL analyzed the data and performed statistical analyses; YZM and WCL drafted the manuscript; LGY, YZB, and QMX provided critical revisions; All authors reviewed and approved the final manuscript.

## Data availability statement

This article presents all the data generated and analyzed in this study. If access to the dataset utilized and/or analyzed in the current research is required, it can be acquired from the corresponding author upon reasonable request.

## Declaration of competing interest

The authors have no competing interests to declare.

## References

[bib0001] Canuti M., Verhoeven J.T.P., Munro H.J., Roul S., Ojkic D., Robertson G.J., Whitney H.G., Dufour S.C., Lang A.S. (2021). Investigating the diversity and host range of novel parvoviruses from North American ducks using epidemiology, phylogenetics, genome structure, and codon usage analysis. Viruses..

[bib0002] Chen C., Wu Y., Li J., Wang X., Zeng Z., Xu J., Liu Y., Feng J., Chen H., He Y., Xia R. (2023). TBtools-II: A "one for all, all for one" bioinformatics platform for biological big-data mining. Mol. Plant.

[bib0003] Chen H., Dou Y., Tang Y., Zhang Z., Zheng X., Niu X., Yang J., Yu X., Diao Y. (2015). Isolation and genomic characterization of a duck-origin GPV-related parvovirus from Cherry Valley ducklings in China. PLoS. One.

[bib0004] Chen H., Dou Y., Tang Y., Zheng X., Niu X., Yang J., Yu X., Diao Y. (2016). Experimental reproduction of beak atrophy and dwarfism syndrome by infection in cherry valley ducklings with a novel goose parvovirus-related parvovirus. Vet. Microbiol..

[bib0005] Cotmore S.F., Agbandje-McKenna M., Canuti M., Chiorini J.A., Eis-Hubinger A.-M., Hughes J., Mietzsch M., Modha S., Ogliastro M., Pénzes J.J., Pintel D.J., Qiu J., Soderlund-Venermo M., Tattersall P., Tijssen P. (2019). ICTV virus taxonomy profile: Parvoviridae. J. General Virol..

[bib0006] Cotmore S.F., Tattersall P. (2014). Parvoviruses: small does not mean simple. Annu Rev. Virol..

[bib0007] Cui H., Pan S., Xu X., Ji J., Ma K., Yao L., Kan Y., Bi Y., Xie Q. (2023). Molecular characteristics of novel chaphamaparvovirus identified in chickens. Poult. Sci..

[bib0008] de Souza W.M., Dennis T., Fumagalli M.J., Araujo J., Sabino-Santos G., Maia F.G.M., Acrani G.O., Carrasco A.d.O.T., Romeiro M.F., Modha S., Vieira L.C., Ometto T., Queiroz L.H., Durigon E.L., Nunes M.R.T., Figueiredo L.T.M., Gifford R.J. (2018). Novel parvoviruses from wild and domestic animals in Brazil provide new insights into parvovirus distribution and diversity. Viruses..

[bib0009] Delabouglise A., Thanh N.T.L., Xuyen H.T.A., Nguyen-Van-Yen B., Tuyet P.N., Lam H.M., Boni M.F. (2020). Poultry farmer response to disease outbreaks in smallholder farming systems in southern Vietnam. Elife.

[bib0010] Gržinić G., Piotrowicz-Cieślak A., Klimkowicz-Pawlas A., Górny R.L., Ławniczek-Wałczyk A., Piechowicz L., Olkowska E., Potrykus M., Tankiewicz M., Krupka M., Siebielec G., Wolska L. (2023). Intensive poultry farming: A review of the impact on the environment and human health. Sci. Total Environ..

[bib0011] Li Y., Gordon E., Idle A., Altan E., Seguin M.A., Estrada M., Deng X., Delwart E. (2020). Virome of a feline outbreak of diarrhea and vomiting includes bocaviruses and a novel chapparvovirus. Viruses..

[bib0012] Martin D.P., Murrell B., Golden M., Khoosal A., Muhire B. (2015). RDP4: Detection and analysis of recombination patterns in virus genomes. Virus. Evol..

[bib0013] Palinski R.M., Mitra N., Hause B.M. (2016). Discovery of a novel parvovirinae virus, porcine parvovirus 7, by metagenomic sequencing of porcine rectal swabs. Virus. Genes..

[bib0014] Palya V., Zolnai A., Benyeda Z., Kovács E., Kardi V., Mató T. (2009). Short beak and dwarfism syndrome of mule duck is caused by a distinct lineage of goose parvovirus. Avian Pathol..

[bib0015] Pang Y., Wang H., Girshick T., Xie Z., Khan M.I. (2002). Development and application of a multiplex polymerase chain reaction for avian respiratory agents. Avian Dis..

[bib0016] Pénzes J.J., Söderlund-Venermo M., Canuti M., Eis-Hübinger A.M., Hughes J., Cotmore S.F., Harrach B. (2020). Reorganizing the family Parvoviridae: a revised taxonomy independent of the canonical approach based on host association. Arch. Virol..

[bib0017] Soliman M.A., Erfan A.M., Samy M., Mahana O., Nasef S.A. (2020). Detection of novel goose parvovirus disease associated with short beak and dwarfism syndrome in commercial ducks. Animals.

[bib0018] Tamura K., Stecher G., Kumar S., Battistuzzi F.U. (2021). MEGA11: Molecular Evolutionary Genetics Analysis Version 11. Mol. Biol. Evol..

[bib0019] Tijssen P., Pénzes J.J., Yu Q., Pham H.T., Bergoin M. (2016). Diversity of small, single-stranded DNA viruses of invertebrates and their chaotic evolutionary past. J. Invertebr. Pathol..

[bib0020] Wan C., Liu R., Chen C., Cheng L., Shi S., Fu G., Chen H., Fu Q., Huang Y. (2019). Novel goose parvovirus in domestic Linwu sheldrakes with short beak and dwarfism syndrome. Transbound. Emerg. Dis..

[bib0021] Yang J., Tian J., Tang Y., Diao Y. (2018). Isolation and genomic characterization of gosling gout caused by a novel goose astrovirus. Transbound. Emerg. Dis..

[bib0022] Yang S., Liu Z., Wang Y., Li W., Fu X., Lin Y., Shen Q., Wang X., Wang H., Zhang W. (2016). A novel rodent chapparvovirus in feces of wild rats. Virol. J..

[bib0023] Yinda C.K., Ghogomu S.M., Conceição-Neto N., Beller L., Deboutte W., Vanhulle E., Maes P., Van Ranst M., Matthijnssens J. (2018). Cameroonian fruit bats harbor divergent viruses, including rotavirus H, bastroviruses, and picobirnaviruses using an alternative genetic code. Virus. Evol..

[bib0024] Yu K., Ma X., Sheng Z., Qi L., Liu C., Wang D., Huang B., Li F., Song M., Fenwick B.W. (2016). Identification of goose-origin parvovirus as a cause of newly emerging beak atrophy and dwarfism syndrome in ducklings. J. Clin. Microbiol..

[bib0025] Zhao Q., Zhao R., Sun Y., Ji L., Xi Y., Wang X., Shen Q., Ji L., Wang Y., You Z., Yang S., Zhang W. (2023). Identification of multiple novel viruses in fecal samples of black-necked cranes using viral metagenomic methods. Viruses..

[bib0026] Zhu J., Yang Y., Zhang X., Chen B., Liu G., Bao E. (2022). Characterizing two novel goose parvoviruses with different origins. Transbound. Emerg. Dis..

